# Efficacy and safety of Qing Zhu Granules for acute gouty arthritis with dampness-heat obstruction syndrome: study protocol for a phase 3, multicenter, randomized, double-blind, placebo-controlled trial

**DOI:** 10.3389/fmed.2026.1839780

**Published:** 2026-07-28

**Authors:** Xinyao Zhou, Fengmei Lian, Ting Zhao, Quan Jiang, Yan Zhao

**Affiliations:** 1 Department of Rheumatology, Guang'anmen Hospital, China Academy of Chinese Medical Sciences, Beijing, China; 2 GCP Office, Guang'anmen Hospital, China Academy of Chinese Medical Sciences, Beijing, China; 3 Department of Rheumatology and Immunology, Peking Union Medical College Hospital, Chinese Academy of Medical Sciences, Beijing, China; 4 National Clinical Research Center for Dermatologic and Immunologic Diseases (NCRC-DID), Beijing, China; 5 State Key Laboratory of Complex Severe and Rare Diseases, Beijing, China; 6 Key Laboratory of Rheumatology and Immuno-disease, Ministry of Education, Beijing, China

**Keywords:** acute pain, gouty arthritis, Qing Zhu Granules, randomized controlled trial, traditional Chinese medicine

## Abstract

Although standard therapies for acute gouty arthritis, such as nonsteroidal anti-inflammatory drugs, colchicine, and glucocorticoids demonstrate efficacy, their clinical application is limited by potential adverse reactions, which remains an unresolved issue. Qing Zhu Granules is a traditional Chinese medicine (TCM) compound preparation used for the treatment of primary acute gouty arthritis with dampness-heat obstruction syndrome. This multicenter, randomized, double-blind, placebo-controlled phase 3 clinical trial conducted in China aimed to evaluate the efficacy and safety of Qing Zhu Granules. Patients who met the Western medical diagnostic criteria for gout, met the TCM syndrome differentiation criteria for dampness-heat obstruction syndrome, had an acute attack duration ≤48 h, and a visual analog scale (VAS) score of joint pain ≥ 40 mm in the current attack were included. Participants were randomly assigned in a 1:1 ratio to the Qing Zhu Granules group or the placebo group, with an oral dose of 6 g, administered 3 times a day, for a treatment course of 3 days. During the treatment period, diclofenac sodium enteric-coated tablets could be used as rescue medication in case of intolerable pain. The primary endpoint was the change in joint pain VAS score (0–100 mm) from baseline at 72 h after treatment. The secondary endpoints included changes in joint pain VAS score from baseline at 24 and 48 h after treatment, changes in the total score and each individual item score of the TCM syndrome rating scale from baseline, changes in C-reactive protein levels from baseline at 72 h after treatment, total dose and frequency of rescue medication within 72 h of treatment, and the interval between the first use of rescue medication and the first use of the study drug after treatment initiation.

**Clinical trial registration:**

https://clinicaltrials.gov/, identifier NCT06068478.

## 1 Introduction

Gout is the most common inflammatory arthritis, affecting an estimated 1–3% of the population in China (1, 2). Acute gout flares can cause severe pain, limited mobility, and functional disability, seriously impairing the patient’s health-related quality of life (3). In the acute phase, the core treatment goal is to quickly control joint inflammation and relieve pain (2). Currently, commonly used clinical drugs include nonsteroidal anti-inflammatory drugs (NSAIDs), colchicine, and glucocorticoids (oral or injectable) (2, 4). However, their clinical application is limited by potential adverse effects. Specifically, NSAIDs are contraindicated in patients with active gastrointestinal bleeding or perforation and may cause nephrotoxicity, necessitating close monitoring of renal function. Colchicine is commonly associated with gastrointestinal adverse reactions such as diarrhea, abdominal pain, nausea, and vomiting, and may cause hepatotoxicity, nephrotoxicity, and myelosuppression. Glucocorticoids may induce adverse reactions such as hypertension, hyperglycemia, hyperlipidemia, sodium and water retention, increased risk of infection, gastrointestinal bleeding, and osteoporosis (2). In recent years, interleukin-1 (IL-1) inhibitors have attracted increasing attention with the development of biological agents (4–7). Nevertheless, firsekibart, introduced in 2025, remains the only IL-1 inhibitor currently available in China, and large-scale real-world evidence regarding its use is still lacking (8). Consequently, there is an urgent clinical need for more safe and effective treatment options.

Traditional Chinese Medicine (TCM) has been employed for centuries in the management of gout in China (9). According to TCM theory, acute gout is primarily characterized by dampness-heat obstruction syndrome, with main clinical manifestations including acute onset of severe joint swelling, redness, heat, and pain. Secondary manifestations encompass limited joint movement, fever, restlessness, tongue conditions of red with yellow, greasy or thick yellow coating, and the pulse conditions of string-like, slippery, or rapid. The primary treatment principle is clearing heat and eliminating dampness, promoting blood circulation, and dredging collaterals (10). Numerous randomized clinical trials have confirmed the efficacy and safety of TCM in the treatment of acute gout attacks, e.g., external preparations such as Qingbi granule (11), Qingpeng ointment (12), Qingluo san (13), Qinpi Tongfeng Formula (14) and Xin Huang Pian (15), as well as the oral preparation Huzhang granule (16). Previous clinical trials have demonstrated that these drugs, either used alone or in combination with NSAIDs, improved gout-related indicators such as the pain VAS score. Existing evidence supports further research on TCM to enhance therapeutic efficacy and expand its clinical value.

Qing Zhu Granules, derived from an empirical formula of TCM master Lu Zhizheng, were formulated as a granular preparation by Tasly Pharmaceutical Group Co., Ltd. This formulation consists of nine TCM herbs, i.e., Qing Feng Teng (*Sinomenii Caulis*), Fu Chao Cang Zhu (*Rhizoma Atractylodis Lanceae*), Huang Bo (*Phellodendri Chinensis Cortex*), Fu Chao Zhi Shi (*Aurantii Fructus Immaturus*), Hu Zhang (*Polygoni Cuspidati Rhizoma Et Radix*), Dan Shen (*Salviae Miltiorrhizae Radix Et Rhizoma*), Yi Mu Cao (*Leonuri Herba*), Mian Bi Xie (*Dioscoreae Spongiosae Rhizoma*) and Zao Jiao Ci (*Gleditsiae Spina*) (Table 1). It exerts the effects of clearing heat and eliminating dampness, promoting blood circulation and dredging collaterals, reducing swelling and relieving pain, in line with the TCM compatibility theory.

**Table 1:** Compositions of Qing Zhu Granules.

Chinese name	Latin name	Amount (g)	Category of medicine^a^
Qing Feng Teng	*Sinomenii Caulis*	3.97	Sovereign
Fu Chao Cang Zhu	*Rhizoma Atractylodis Lanceae*	1.99	Minister
Huang Bo	*Phellodendri Chinensis Cortex*	1.32	Minister
Fu Chao Zhi Shi	*Aurantii Fructus Immaturus*	1.59	Assistant
Hu Zhang	*Polygoni Cuspidati Rhizoma Et Radix*	1.99	Assistant
Dan Shen	*Salviae Miltiorrhizae Radix Et Rhizoma*	1.59	Assistant
Yi Mu Cao	*Leonuri Herba*	1.99	Assistant
Mian Bi Xie	*Dioscoreae Spongiosae Rhizoma*	1.99	Assistant
Zao Jiao Ci	*Gleditsiae Spina*	1.58	Courier

a Traditional Chinese medicine generally applies the “sovereign-minister-assistant-courier” rule: sovereign medicines act as the main constituents providing therapeutic effects; minister medicines assist the sovereign medicines in boosting the therapeutic effects; assistant medicines treat the related symptoms or reduce the toxic effects of sovereign and minister medicines; courier medicines direct the other medicines to the diseased organ or harmonize their effects.

In addition to its theoretical basis in TCM, Qing Zhu Granules have demonstrated promising pharmacological activities in preclinical studies. Qing Zhu Granules exerted significant anti-inflammatory effects in multiple experimental animal models, including both acute inflammatory and chronic proliferative inflammatory conditions, with evidence of a dose–response relationship. The formulation also demonstrated notable analgesic effects against chemical stimulus-induced, thermal stimulus-induced, and damp-heat stimulus-induced pain. In particular, its analgesic activity in the rat hot-water tail-flick model exhibited both dose-dependent and time-dependent characteristics (17). Additionally, constituent herbs of the formula have been shown to suppress pivotal mediators of gout-related inflammation, including interleukin (IL)-6, IL-1*β*, prostaglandin E₂, nuclear factor-κB, and tumor necrosis factor-*α* (18–20). These findings bridge TCM theory with contemporary biomedical understanding, offering mechanistic plausibility, particularly in the context of the central pathogenic role of the NOD-like receptor thermal protein domain associated protein 3 (NLRP3) inflammasome-IL-1*β* axis in acute gouty arthritis (18–20).

For the control group, this trial adopts a placebo-controlled design. According to the General Principles for Clinical Research of New Traditional Chinese Medicine, primary acute gouty arthritis is a self-limiting disease, and short-term non-treatment is generally not expected to adversely affect long-term prognosis (21). Therefore, a placebo control was considered scientifically and ethically acceptable for evaluating the efficacy and safety of Qing Zhu Granules. To minimize participant risk and ensure adequate symptom control, rescue therapy with diclofenac sodium enteric-coated tablets was permitted during the treatment period in cases of intolerable pain, and all rescue medication use was prospectively recorded. In addition, participants underwent close safety monitoring throughout the study, including adverse event assessments, laboratory testing, physical examinations, vital signs measurements, and electrocardiographic evaluations.

The primary objective of this study was to evaluate the efficacy and safety of Qing Zhu Granules in the treatment of primary acute gouty arthritis with dampness-heat obstruction syndrome, and secondary objectives included the improvement of Qing Zhu Granules on other clinical symptoms and inflammation in patients.

## 2 Methods

### 2.1 Study design and participants

This was a multicenter, randomized, double-blind, placebo-controlled phase 3 clinical trial conducted at 27 research centers across 14 provinces or municipalities in China, with the leading participating center being Guang’anmen Hospital, China Academy of Chinese Medical Sciences. The trial was conducted from November 7, 2023, to April 2, 2025, and is currently in the data analysis phase.

Information about the study was disseminated through participant recruitment notices, which were released after approval by the Ethics Committee (Ethics Approval Date: June 20, 2023; Ethics Approval Number: 2023-103-YW), and participant recruitment was conducted using a competitive enrollment strategy. Patients meeting the diagnostic criteria for primary acute gouty arthritis with dampness-heat obstruction syndrome were included. Investigators fully and comprehensively explained the details of the clinical trial to patients, their legal guardians or impartial witnesses, including the trial’s purpose, procedures, potential benefits and risks, as well as the rights and obligations of participants. Patients were also informed that they had the right to discontinue the study drug at any time. Patients were formally enrolled in the clinical trial after signing the written informed consent form and meeting the inclusion and exclusion criteria through screening. The complete list of inclusion and exclusion criteria is detailed in Table 2. This trial was conducted in accordance with the Declaration of Helsinki and Good Clinical Practice guidelines. The protocol and all amendments were approved by the institutional review board of the leading participating center.

**Table 2:** Inclusion and exclusion criteria.

Inclusion criteria	Exclusion criteria
To be eligible for enrollment, participants should meet all the following criteria during screening: 18–70 years (inclusive) of age, regardless of gender. Western medical diagnostic criteria for gout (refer to the 2015 ACR/EULAR gout classification criteria) (29). TCM syndrome differentiation criteria for dampness-heat obstruction syndrome. Acute attack duration ≤ 48 h. Joint pain VAS score ≥ 40 mm in the attack. Voluntary participation in this clinical trial and signing of the informed consent form.	Participants should be excluded if meeting any of the following criteria: Secondary gouty arthritis (including gout caused by other diseases or drugs). Pain symptoms caused by other diseases that, according to the investigator’s judgment, might affect the safety or efficacy evaluation of the trial. Complications with severe diseases of the motor, digestive, respiratory, urinary, reproductive, endocrine, immune, nervous, circulatory systems or mental illnesses, etc., which, according to the investigator’s judgment, might affect the safety or efficacy evaluation of the trial. Abnormal liver function (aspartate aminotransferase or alanine aminotransferase >2 × upper limit of normal) or abnormal kidney function (serum creatinine > upper limit of normal). Populations for whom VAS evaluation is not applicable, such as individuals with severe deficits in abstract thinking, visual or writing functions, or those administered sedatives, etc. Uric acid-lowering therapy with stable use of uric acid-lowering drugs for at least 2 weeks before randomization. After the onset of the gout attack, use of traditional Chinese medicines, chemical drugs (including but not limited to colchicine, glucocorticoids, and adrenocorticotropic hormone), biological agents (including but not limited to IL-1 and TNF-*α* inhibitors), or non-pharmacological treatments (including but not limited to acupuncture and ice application) that are effective for gout. After the onset of the gout attack, use of NSAIDs (including but not limited to aspirin, acetaminophen, loxoprofen, ibuprofen, and diclofenac sodium) within 5 half-lives of the drug before randomization. Known allergy to any component of the study drug. Contraindications to diclofenac sodium enteric-coated tablets. Fertility plans within 3 months from the study’s start to its end (males or females). Pregnancy or lactation in women. Participation in other clinical trials within the past 1 month. Other conditions deemed inappropriate for enrollment by the investigators.

ACR, American College of Rheumatology; EULAR, European Alliance of Associations for Rheumatology; TCM, traditional Chinese medicine; VAS, visual analog scale; IL-1, interleukin-1; TNF-α, tumor necrosis factor-alpha; NSAIDs, nonsteroidal anti-inflammatory drugs.

### 2.2 Randomization and blinding

This trial adopted a block randomization method to randomly assign participants to the Qing Zhu Granules and placebo groups in a 1:1 ratio. A biostatistician generated a random sequence using the SAS statistical software (version 9.4). A centralized interactive web response system (IWRS) was used for random number assignment and drug management. Researchers not involved in the trial affixed the package numbers of the study drug and placebo to the labels to ensure allocation concealment.

A double-blind design was implemented, with blinding applied to the sponsor, investigators, relevant research personnel, and participants. The placebo was consistent with the study drug in terms of color, odor, taste, shape, texture, and other characteristics, and an evaluation report for the placebo was obtained. Investigators should not unblind the study drug unless it is necessary to obtain information about the study drug for the medical treatment of participants. In the event of a medical emergency requiring immediate identification of the drug administered to a participant, every effort should be made to notify the principal investigator and relevant personnel of the sponsor. Emergency unblinding may be performed only after obtaining approval from the principal investigator to confirm the administered drug. If contact with the sponsor cannot be made before unblinding, the sponsor must be contacted within 24 h after unblinding. Investigators should record the time, location, and reason for unblinding.

### 2.3 Study intervention

Patients in the study group received Qing Zhu Granules, and those in the control group received a matching placebo. The dose and administration schedule for both groups were 1 sachet (6 g) each time, 3 times a day, orally, for a treatment course of 3 days (Table 1). Dose adjustment was not allowed during the study period. Criteria for treatment discontinuation included withdrawal of informed consent by participants, occurrence of adverse events during the trial that required discontinuation of medication as judged by investigators, pregnancy in female participants, or other specific reasons.

Qing Zhu Granules were manufactured on a Good Manufacturing Practice (GMP)-compliant production line. Product quality was established in accordance with the Chinese Pharmacopeia (2025 Edition) and the Technical Guidelines for the Study of Quality Standards of New Traditional Chinese Medicines (Trial) (22, 23). Quality control included thin-layer chromatography identification of representative herbal components, as well as examinations of moisture content, particle size, dissolution, fill weight variation, and microbial limits. Quantitative determination of sinomenine and berberine hydrochloride demonstrated good inter-batch consistency across manufactured batches, supporting the stability and standardization of the study drug.

The placebo contained dextrin, corn starch, caramel color, sunset yellow, lemon yellow, sucrose octoacetate, and stevioside, and was formulated to match Qing Zhu Granules in appearance, color, taste, texture, and dissolution characteristics without containing any active herbal ingredients.

During the treatment period, diclofenac sodium enteric-coated tablets (25 mg/tablet) could be used as rescue treatment as needed in case of intolerable pain. The medication should be administered in accordance with the prescribing information, with a maximum of 6 tablets per day. Administration was prohibited within 12 h before the assessment of the primary efficacy endpoint (72-h joint pain VAS score). For concomitant treatments, participants were allowed to continue using uric acid-lowering drugs that had been stably used for at least 2 weeks before screening. Adjustment of the type or dosage of such therapy was prohibited during the trial unless necessary; any necessary adjustments should be recorded. Stable concomitant medications for other pre-existing diseases were permitted, as were treatments for adverse events. The use of any other therapies for pain, gout, or hyperuricemia was prohibited, including colchicine, allopurinol, febuxostat, NSAIDs, IL-1 inhibitors, glucocorticoids, TCMs, as well as acupuncture or ice application.

Investigators provided dietary and lifestyle guidance, including restricting the intake of alcohol, fructose, and high-purine foods, appropriately increasing water and vegetable intake, maintaining a regular diet, avoiding strenuous exercise and sudden cold exposure, controlling weight, and maintaining regular exercise and work-rest schedules. This study adopted a short-term oral administration regimen, and good participant compliance was expected. The quantities of the study drugs distributed, taken, and returned were recorded in detail to assess compliance. Compliance rate = (actual number of sachets taken/9 sachets) × 100%.

### 2.4 Procedure

The study schedule is shown in Table 3. In case participants failed to complete visits as planned, every effort should be made to contact them. If contact with participants was unsuccessful after ≥3 attempts through various methods, they were considered lost to follow-up, and the process of attempted contact should be clearly recorded in the source documents. A distinction should be made between voluntary withdrawal of informed consent by participants and loss to follow-up during documentation.

**Table 3:** Study procedure.

Study procedure	Screening/baseline period (−24 ~ 0 h)	Treatment period (0 ~ 72 h)		
	−24 ~ 0 h	24 ± 2 h	48 ± 2 h	72 ± 6 h or early withdrawal^a^
	Visit 1			Visit 2
Sign informed consent form	X			
Assign screening number	X			
Baseline characteristics	X			
Vital signs	X			X
Physical examination	X			X
Western medical diagnosis and TCM syndrome differentiation	X			
Joint ultrasound or dual-energy CT	X			
Blood routine	X			X
C-reactive protein	X			X
Blood biochemistry	X			X
Urine routine	X			X
Urine pregnancy test	X			
12-lead electrocardiogram	X			X
TCM syndrome grading scale score	X			X
VAS score of joint pain	X	X	X	X
Review inclusion and exclusion criteria	X			
Randomization and enrollment	X			
Record adverse events		X	X	X
Record concomitant treatments	X	X	X	X
Distribute study drugs and rescue medication	X			
Collect unused study drugs and rescue medication				X
Distribute diary cards	X			
Collect diary cards				X

a For patients who withdraw early, efficacy and safety data should be collected as much as possible.

CT, computed tomography; TCM, traditional Chinese medicine; VAS, visual analog scale.

### 2.5 Outcomes

The primary endpoint was the change in VAS (0–100 mm) score of joint pain from baseline at 72 h after treatment. VAS scores were recorded by participants in a diary card and subsequently measured and recorded by the investigators.

Secondary endpoints included: (1) Changes in VAS score of joint pain from baseline at 24 and 48 h after treatment, which was used to evaluate the efficacy of Qing Zhu Granules at different time points and to assess the rapidity of treatment response. (2) Proportions of participants with a one-grade decrease in VAS score at 24 h, 48 h, and 72 h after treatment. VAS scores were divided into three grades ([0, 40], [40, 70], and [70, 100]) to assess the proportions of participants achieving effective pain relief at different time points. (3) Proportion of participants with a VAS score ≤10 mm at 72 h after treatment, which was used to evaluate the proportion of participants achieving pain relief after 72 h of treatment. (4) Proportions of participants with a 50% decrease in VAS score at 24 h, 48 h, and 72 h after treatment, which were used to assess the proportions of participants achieving effective joint pain relief at different time points. (5) Changes in the total score and each individual item score of the TCM syndrome rating scale from baseline, as well as the disappearance rate of each individual item at 72 h after treatment. (6) Change in C-reactive protein (CRP) levels from baseline. (7) Total dosage and administration frequency of the rescue medication (diclofenac sodium enteric-coated tablets) within 72 h of treatment; participants were required to accurately record the time and dose of each medication intake on a diary card, as well as the interval between the first use of the rescue medication and the first use of the study drug after treatment initiation. TCM syndromes were evaluated by the investigator using the grading scale (Table 4). To ensure that different investigators had a full understanding of the scale, unified training on the clinical trial protocol was provided to personnel participating in the clinical trial before its implementation.

**Table 4:** Traditional Chinese medicine syndrome rating scale.

Symptoms	Description
Joint pain	0 point: No joint pain or pain has disappeared 2 points: Pain in 1 joint, mild pain that is tolerable, or pain only occurs with fatigue or during weather changes, basically not affecting work 4 points: Pain in 2–3 joints, or moderate pain that affects both work and rest 6 points: Pain in more than 3 joints, or severe pain that is intolerable, seriously affecting rest and work, and even requiring the use of painkillers
Joint swelling	0 point: No joint swelling or swelling has disappeared 2 points: Swelling in 1 joint, mild swelling with shallow skin texture, and bone landmarks of the joint are still visible 4 points: Swelling in 2–3 joints, or moderate swelling with basically invisible skin texture and indistinct bone landmarks 6 points: Swelling in more than 3 joints, or severe swelling with tight skin and invisible bone landmarks
Skin temperature and color	0 points: Normal skin temperature and color at the painful joint site 1 point: Mildly increased skin temperature at the painful joint site, with normal skin color 2 points: Significantly increased skin temperature at the painful joint site, with red skin color
Limited joint mobility	0 points: Normal joint movement 1 point: Mildly limited joint movement 2 points: Significantly limited joint movement or even stiffness
Fever	0 points: Axillary temperature <37.3°C 1 point: Axillary temperature ≥37.3°C
Restlessness	0 points: None 1 point: Present

The TCM syndrome rating scale was developed and finalized through expert consensus at the Initial Investigator Meeting for Qing Zhu Granules (May 21, 2023) and was prospectively incorporated into the study protocol.

The safety evaluation included assessment of adverse events combined with vital signs, physical examinations, laboratory results, and 12-lead electrocardiograms. Laboratory assessments included blood routine examinations (white blood cell count, red blood cell count, hemoglobin, platelet count, neutrophil count, eosinophil count, basophil count, and lymphocyte count), blood biochemistry tests (alanine aminotransferase, aspartate aminotransferase, alkaline phosphatase, gamma-glutamyl transferase, total bilirubin, direct bilirubin, creatinine, urea or blood urea nitrogen, and uric acid), and urinalysis (protein, urine glucose, ketone bodies, white blood cells, red blood cells, and pH). Adverse events were coded using the Medical Dictionary for Regulatory Activities (MedDRA; version 26.0 or higher) and graded according to the Common Terminology Criteria for Adverse Events (CTCAE) version 5.0. Each adverse event was assessed for causality, severity (Grade 3 or higher), and classification as a serious adverse event, as well as whether it led to treatment discontinuation, trial withdrawal, or death. Previous clinical trials have shown that Qing Zhu Granules have good overall safety; in addition, its compatibility based on the “sovereign, minister, assistant and courier” theory helps achieve synergistic efficacy and reduced toxicity (24). Nonetheless, ongoing vigilance for potential adverse event risks remains warranted. The most commonly reported adverse reactions to date include diarrhea and mild elevations in liver enzymes; corresponding safety management strategies are detailed in Table 5.

**Table 5:** Risk management.

Potential risks	Risk management strategies
Gastrointestinal system (nausea, vomiting, stomach pain, diarrhea)	Advise patients to supplement various nutrients during the trial and prohibit the consumption of moldy, rough, strong, and spicy foods. In cases of nausea, vomiting, or diarrhea, monitor frequency and the volume, color, and consistency of emesis/stool, while ensuring sufficient fluid intake. Contact the investigator to identify the cause and enter into the record. If necessary, consult gastroenterologists for diagnosis and treatment.
Liver and kidney injury	Exclude the following patients based on risk considerations: Patients with abnormal liver function (aspartate aminotransferase or alanine aminotransferase > 2 × upper limit of normal) or abnormal kidney function (serum creatinine > upper limit of normal) Patients with complicated severe diseases of the motor, digestive, respiratory, urinary, reproductive, endocrine, immune, nervous, circulatory systems, or mental illnesses, etc. Based on the investigator’s assessment, frequencies of liver and kidney function tests may be increased if necessary. Advise the participants to use drugs that significantly affect liver function with caution. If necessary, consult specialist physicians for diagnosis and treatment.
Abnormal blood routine examination	Based on the investigator’s assessment, frequencies of blood routine tests may be increased if necessary. If necessary, consult specialist physicians for diagnosis and treatment.
Allergic Reactions (rash)	In case of allergic reactions, advise subjects to contact the investigating physicians promptly and document the reactions. After evaluation by investigators, advise subjects to visit a hospital if necessary.

### 2.6 Sample size calculation

A prior study of Qing Zhu Granules evaluated the numeric pain rating scale (NRS) score. On day 2, the difference in joint pain NRS score from baseline was −1.34 cm in the trial group versus −0.99 cm in the placebo group, with a difference of −0.35 cm between the Qing Zhu Granules and placebo groups. Through conversion between the VAS and NRS scales, the difference in joint pain VAS score from baseline on day 2 was −13.4 mm in the Qing Zhu Granules group versus −9.9 mm in the placebo group, with a difference of −3.5 mm between the two groups. The primary endpoint of the current study was the change in joint pain VAS score from baseline at 72 h after treatment. Since no prior clinical data were available for the 72-h timepoint, and considering that the treatment effect may increase over time, a between-group difference of −3.8 mm was estimated for sample size calculation. Assuming a standard deviation (SD) of 13.5 mm, a significance level *α* = 0.05 (two-sided), and a type II error *β* = 0.2 (power 80%), with a 1:1 randomization ratio, a total of 398 participants were required for both groups. Considering an approximate 15% dropout rate, the estimated final sample size was 472 participants, including 236 participants each in the trial and placebo groups.

### 2.7 Statistical analysis

Efficacy analyses were performed in both the intention-to-treat (ITT) population, comprising all randomized participants, and the modified ITT (mITT) population, defined as randomized participants who received at least one dose of the study drug and had at least one post-baseline efficacy assessment. Safety was assessed in the safety set (SS), which included all randomized participants who received at least one dose of study drug and had at least one safety evaluation.

The SAS software (version 9.4) was used for analysis. All statistical tests and confidence intervals (CI) were two-sided, with a statistically significant significance level set at two-sided 0.05; a two-sided 95% CI was used for parameter estimation. For continuous variables, descriptive statistics included the number of participants, number of missing participants, mean, standard deviation, minimum value, 25th percentile (P25), median, 75th percentile (P75), and maximum value. Categorical variables were described by the frequency counts and percentages. Analysis of Covariance (ANCOVA) was used for continuous variables, with group as the fixed factor and baseline joint pain VAS score as the covariate, to assess the least squares mean (LSM) between-group difference and its two-sided 95% CI. Between-group comparisons used the Pearson χ^2^ test or the Fisher’s exact test, and the 95% CI for the between-group rate difference was calculated by the Newcombe method. When examining the impact of the center factor, the Cochran–Mantel–Haenszel (CMH) test was used for between-group comparisons, and the 95% CI for the between-group rate difference was estimated by the stratified Newcombe method based on weights estimated by the Mantel–Haenszel (MH) method. The unstratified Log-rank test was used to calculate the *p*-value for each between-group comparison, and the unstratified Cox proportional hazards model was used to estimate the hazard ratio (HR) between groups and the corresponding 95% CI. Based on the Missing at Random (MAR) assumption, multiple imputation was used for missing VAS score data, and other missing values were not imputed.

Exploratory subgroups include baseline joint pain intensity (≥70 mm vs. <70 mm), age (>40 years vs. ≤40 years), and background urate-lowering therapy status (stable urate-lowering therapy use at baseline vs. no urate-lowering therapy use at baseline). Given the short treatment duration (3 days), brief follow-up period, and the favorable efficacy and safety profile observed in previous studies of Qing Zhu Granules, no interim analysis was planned for this study. Details for the sensitivity analysis are provided in Table 6.

**Table 6:** Sensitivity analysis.

Sensitivity analysis	Descriptions
Sensitivity Analysis 1	Between-group comparison method: ANCOVA was used, with the change in joint pain VAS score from baseline at 72 h after treatment as the dependent variable, group as the fixed factor variable, and baseline joint pain VAS score as the covariate to calculate the LSM difference between the two groups and the related 95% CI.
	Missing data handling: Missing data were imputed using the LOCF method.
Sensitivity Analysis 2	Between-group comparison method: MMRM was used, with group, visit, and the interaction term between group and visit as fixed factor variables, and baseline joint pain VAS score as the covariate, to calculate the LSM difference between the two groups and the related 95% CI.
	Missing data handling: No imputation for missing data
Sensitivity Analysis 3	Between-group comparison method: ANCOVA was used, with the change in joint pain VAS score from baseline at 72 h after treatment as the dependent variable, group as the fixed factor variable, and baseline joint pain VAS score as the covariate, to calculate the LSM difference between the two groups and the related 95% CI.
	Missing data handling: The tipping-point approach was used to identify the tipping point where the conclusion reverses.
Sensitivity Analysis 4	Exclude participants with a medication adherence percentage outside the range of 80–120% for between-group comparisons. Between-group comparison method: ANCOVA was used, with the change in joint pain VAS score from baseline at 72 h after treatment as the dependent variable, group as the fixed factor variable, and baseline joint pain VAS score as the covariate, to calculate the LSM between-group difference and the related 95% CI.
	Missing data handling: No imputation for missing data.

ANCOVA, analysis of covariance; VAS, visual analog scale; LSM, least squares mean; CI, confidence interval; LOCF, last observation carried forward; MMRM, mixed-effects model for repeated measures.

### 2.8 Data management and monitoring

Data management and analyses were performed by Guangzhou Keli Medical Research Co., Ltd. This study used an Electronic Data Capture (EDC) System for data management. Investigators retained all trial data, including confirmation of all involved participants (to effectively verify different records, such as original hospital records), all original signed informed consent forms, and detailed records of drug distribution and return. Investigators should retain the data for at least 5 years after the study drug was approved for marketing or 5 years after the termination of the clinical trial.

The sponsor (Tasly Pharmaceutical Group Co., Ltd.) and the contract research organization (Beijing Excellence Future International Pharmaceutical Technology Development Co., Ltd.) formulated an overall project monitoring plan and a quality control plan. Monitors conducted regular monitoring in accordance with standard operating procedures and the monitoring plan. Without violating confidentiality principles and relevant regulations, monitors, auditors, Ethics Committee members, and drug regulatory authorities may inspect the original medical records of participants to verify the clinical trial’s process and data. Except as permitted by regulations, records related to participants’ participation in the clinical trial should be kept confidential and should not be disclosed. Participants’ clinical trial data were stored in the relevant management department of the hospital. If trial results are published, participants’ personal identification information will remain confidential.

### 2.9 Protocol amendments

Any revisions related to the study protocol should be jointly determined through consultation between the sponsor and the clinical research institution, implemented in accordance with the requirements of the *Technical Guiding Principle on Protocol Amendments During Drug Clinical Trials* (*Trial Implementation*) (25), and take effect after obtaining approval from the Ethics Committee or completing the filing. All protocol revisions must be recorded in a protocol amendment form, which should list the differences between the current and previous versions.

### 2.10 Dissemination

Trial data will be communicated to participating individuals, healthcare professionals, and the general public via publication in peer-reviewed journals and presentations at scientific meetings.

## 3 Discussion

This study comprehensively evaluated the effects of Qing Zhu Granules in rapidly relieving joint pain, improving TCM syndromes, and reducing inflammatory responses through a rigorously designed multicenter, randomized, controlled trial, suggesting that it may provide a promising TCM regimen for the treatment of acute gouty arthritis. Currently, standard treatments for acute gout flares may be accompanied by risks of gastrointestinal, nephrotoxicity, and/or metabolism-related adverse events, resulting in intolerance or contraindications in certain patients (2). Building upon this background, Qing Zhu Granules is expected to serve as a clinical therapeutic agent for patients with acute gouty arthritis. The present study is also aligned with the growing emphasis on clinical value-oriented development of TCM. Recent initiatives have highlighted the importance of addressing unmet clinical needs through rigorous clinical evaluation and the generation of high-quality evidence (26). Given the limitations of existing therapies in some patients with acute gouty arthritis, evaluating the efficacy and safety of Qing Zhu Granules may help clarify its potential clinical value and contribute to evidence-based integration of traditional Chinese medicine into contemporary healthcare practice.

Beyond the TCM framework, the objectives of the present study are consistent with contemporary international gout management strategies. Current American College of Rheumatology guidelines emphasize the importance of early intervention during acute gout flares to achieve rapid symptom control and improve patient outcomes (4). Accordingly, this study enrolled patients within 48 h of flare onset and evaluated pain relief at multiple early time points (24, 48, and 72 h), reflecting the clinical priority of prompt flare management. If proven effective, Qing Zhu Granules may provide an additional therapeutic option for patients requiring rapid symptom relief during acute gout attacks. As this trial was conducted exclusively in China, further studies are needed to evaluate the generalizability of the findings in broader populations. Future international multicenter trials may help establish the role of Qing Zhu Granules in diverse clinical settings and further explore its applicability beyond China.

The primary endpoint of this study was the change in joint pain VAS score from baseline at 72 h after treatment, aiming to evaluate the ability of Qing Zhu Granules to rapidly relieve joint pain and shorten the disease course of acute gouty arthritis. Based on previous studies, we anticipated that the Qing Zhu Granules group would be superior to the placebo group in pain relief. If confirmed, this result would support Qing Zhu Granules as a potential therapeutic option for acute gout attacks. Among existing studies assessing oral TCM preparations, a randomized controlled trial of Huzhang Granule showed that its analgesic efficacy from day 2 to day 5 after administration was comparable to that of etoricoxib, with similar mean reductions in VAS scores (51.22 mm in the Huzhang Granule group vs. 52.00 mm in the etoricoxib group) and a treatment difference of 0.78 mm. This indicated the potential of single TCM herbs in the treatment of acute gout. However, the primary endpoint of the aforementioned trial was analgesic efficacy from days 2 to 5, and the rapidity of pain relief in the immediate post-intervention period (e.g., within 72 h) remains to be improved (16). Through its unique compatibility, Qing Zhu Granules may exert synergistic effects in clearing heat and eliminating dampness, promoting blood circulation and dredging collaterals, thereby providing stronger and more rapid pain relief in the treated patients.

This study has several advantages. Firstly, the adoption of a randomized, double-blind, placebo-controlled design maximized bias control to provide high-level evidence. Secondly, the evaluation dimensions were comprehensive, covering multiple aspects from mechanisms to clinical benefits: for the primary endpoint, the VAS score was a patient-reported outcome that directly reflects patients’ pain perception. Considering the acute onset of gout, where joint pain usually peaks within 24 h after onset, this study selected multiple time points (24 h, 48 h, and 72 h) for assessment, which was consistent with the natural course of the disease (27). In addition, according to the recommendations of clinical trial guidelines for gout, responder analysis should be used to illustrate clinical significance in case the primary endpoint is the mean change in VAS score from baseline (28). Therefore, based on changes in VAS score, this study also conducted assessment by grades, evaluated pain disappearance rates, and performed responder rate analysis, which can more intuitively reflect the impact of treatment on patients’ pain status in terms of severity. The secondary endpoints of this study included TCM syndrome scores and rescue medication use, which may help comprehensively evaluate whether Qing Zhu Granules can reduce the use of NSAIDs while controlling the condition, thereby potentially reducing treatment-related adverse events. Meanwhile, by monitoring C-reactive protein levels, this study preliminarily explored the potential anti-inflammatory effects of Qing Zhu Granules, providing a theoretical basis for its mechanism of action. In addition, the study enrolled participants from 27 research centers across different regions of China (Northern, Southern, Western, Eastern, and Central China), enhancing the generalizability of results. The limitations of this study include the exclusion of patients with intercritical gout. Another limitation of this study is the absence of an active comparator. Future randomized controlled trials comparing Qing Zhu Granules directly with standard therapies are warranted to further define its relative efficacy and safety in clinical practice.

In conclusion, this study is expected to provide scientific evidence for the treatment of acute gouty arthritis with Qing Zhu Granules, potentially providing an alternative therapeutic option for gout, and further demonstrating the value of TCM in the management of chronic diseases.
